# Exogenous melatonin alleviates PEG-induced short-term water deficiency in maize by increasing hydraulic conductance

**DOI:** 10.1186/s12870-020-02432-1

**Published:** 2020-05-14

**Authors:** Yujie Qiao, Jianhong Ren, Lina Yin, Yijian Liu, Xiping Deng, Peng Liu, Shiwen Wang

**Affiliations:** 1 State Key Laboratory of Soil Erosion and Dryland Farming on the Loess Plateau, Institute of Soil and Water Conservation, Northwest A&F University, Yangling, 712100 Shaanxi China; 2 College of Life Sciences, Northwest A&F University, Yangling, 712100 Shaanxi China; 3 Institute of Soil and Water Conservation, Chinese Academy of Sciences & Ministry of Water Resources, Yangling, 712100 Shaanxi China; 4 College of Natural Resources and Environment, Northwest A&F University, Yangling, 712100 Shaanxi China; 5 College of Plant Protection, Shandong Agricultural University, Taian, 271018 China

**Keywords:** Aquaporin, Melatonin, Water deficiency, Whole-plant hydraulic conductance, Root hydraulic conductance

## Abstract

**Background:**

Water deficiency is likely to become more frequent and intense as a result of global climate change, which may severely impact agricultural production in the world. The positive effects of melatonin (MEL) on alleviation drought or osmotic stress-induced water deficiency in plants has been well reported. However, the underlying mechanism of MEL on the detailed process of plant water uptake and transport under water deficiency condition remains largely unknown.

**Results:**

Application of 1 μM MEL led to enhanced tolerance to water deficiency stress in maize seedlings, as evidenced by maintaining the higher photosynthetic parameters, leaf water status and plant transpiration rate. The relatively higher whole-plant hydraulic conductance (K_plant_) and root hydraulic conductance (Lp_r_) in MEL-treated seedlings suggest that exogenous MEL alleviated water deficiency stress by promoting root water absorption. HgCl_2_ (aquaporin inhibitor) treatment inhibit the transpiration rate in MEL-treated plants greater than those of MEL-untreated; after recovery by dithiothreitol (DTT, anti-inhibitor), the transpiration rate in MEL-treated plants increased much higher than those of untreated plants. Moreover, under water deficiency, the transcription level of aquaporin genes was up-regulated by MEL application, and the H_2_O_2_ was less accumulated in MEL-treated root.

**Conclusions:**

Exogenous MEL promoted aquaporin activity, which contributed to the maintaining of Lp_r_ and K_plant_ under short-term water deficiency. The increased water uptake and transport lead to improved water status and thus increased tolerance to PEG-induced short-term water deficiency in maize seedlings.

## Background

Melatonin (N-acetyl-5-methoxytryptamine, MEL), a ubiquitously distributed natural pleiotropy bio-molecule, has been confirmed to exist from prokaryotes to eukaryotes, animals to plants [1, 2]. In plants, the positive effects of MEL on regulating plant growth and development have been widely reported, such as promoting seed germination, regulating root and shoot development and adjusting flowering period [1, 3–5]. It has also been widely reported that MEL is involved in regulating plant responses to various biotic and abiotic stresses, such as improve the resistance of plants to drought, cold, heat, osmotic stress, herbicides, UV irradiation and oxidative stress [5]. Meanwhile, various physiological processes have also been found to be involved in MEL regulating plant stress resistance, including increased antioxidant capacity; delayed leaf senescence, promoted development of root system, maintained chlorophyll content and photosynthetic electron transport systems, promoted photosynthetic rate, and so on [6–8]. Recent transcriptome studies have also demonstrated that plant hormones, such as abscisic acid, auxin and gibberellin are also involved in the signaling pathways of MEL regulating plant stress responses [9].

Drought or osmotic stress-induced water deficiency is one of the most severe abiotic stresses in agricultural production, and MEL has been widely reported to improve plant tolerance to water deficiency stress in various plants [7, 10–13]. Numbers of physiological and biochemical processes have been reported to be involved in MEL-mediated plant water deficiency stress response. Among them, MEL-mediated scavenging of reactive oxygen species by increasing antioxidant capacity has been proved to be an important mechanism for improving plant tolerance to water deficiency stress [12, 14]. It has been reported that exogenous MEL was able to increase not only the activities of many antioxidant enzymes, including catalase, superoxide dismutase, peroxidase and ascorbate peroxidase, but also the level of non-enzymatic antioxidants, including glutathione and ascorbate, as well as the expression level of related genes to increase the antioxidant capacity of drought-stressed plants [15, 16]. In addition, exogenous MEL can also promote the biosynthesis of endogenous melatonin under drought stress, which has been shown to directly donate electrons to scavenge free radicals [17, 18]. MEL can also delay drought-induced leaf senescence by scavenging reactive oxygen species [19]. Rhizosphere application of MEL (10 μM) was found to promote the nitrogen metabolism and proline homeostasis in drought-stressed alfalfa, and finally lead to a higher level of chlorophyll fluorescence and stomatal conductance [6].

The drought and osmotic stress-induced damage in plants is mainly due to the water shortage or the imbalance of plant water absorption and loss. Therefore, maintaining plant water balance by reducing water loss or increasing root water absorption is an essential way for improving the plant tolerance to water deficiency stress [20]. MEL application has been widely shown to improve plant drought and osmotic stress tolerance, but its effect on plant water balance has been largely ignored. Although previous research has reported that MEL could maintain the leaf relative water contents by increasing the osmotic adjustment ability [6], less research has been focused on the function of MEL on plant water balance, especially on the plant water uptake and transport ability.

The overall water uptake and transport ability in plant is represented by the whole-plant hydraulic conductance (K_plant_), which consists of leaf, stem and root hydraulic conductance (Lp_r)_ [21]. In plant water transport system, Lp_r_ is the limited factor of overall plant water uptake under water deficiency condition [22]. Lp_r_ is mainly regulated by aquaporin activity before the change of root morphology and structure, it plays a prominent role in plant response to short-term water deficiency stress [23]. It has been shown that MEL could significantly increase the mRNA expression level of aquaporin and the protein level of aquaporin isoforms in animals [24]. In addition, during the postharvest life in tomato, MEL treatment increased the expression of aquaporin genes with enhanced water loss in tomato fruit [25]. These studies suggest that MEL may also be able to increase water absorption capacity by regulating aquaporins in plant roots.

Therefore, the aim of the present study was to test the hypothesis that MEL improves the plant drought resistance by regulating the K_plant_. Maize seedlings were grown in hydroponic solution with and without MEL (1 μM), and 10% polyethylene glycol (PEG) 6000 was used for inducing water deficiency. The photosynthesis, leaf water status and plant transpiration were determined. K_plant_, root xylem osmotic potential, Lp_r_ and the expression level of aquaporin genes were also investigated to exploring the regulation of plant hydraulic conductance by exogenous MEL under water deficiency condition.

## Results

### Effects of MEL application on the photosynthetic capacity under water deficiency stress

**Fig. 1:**
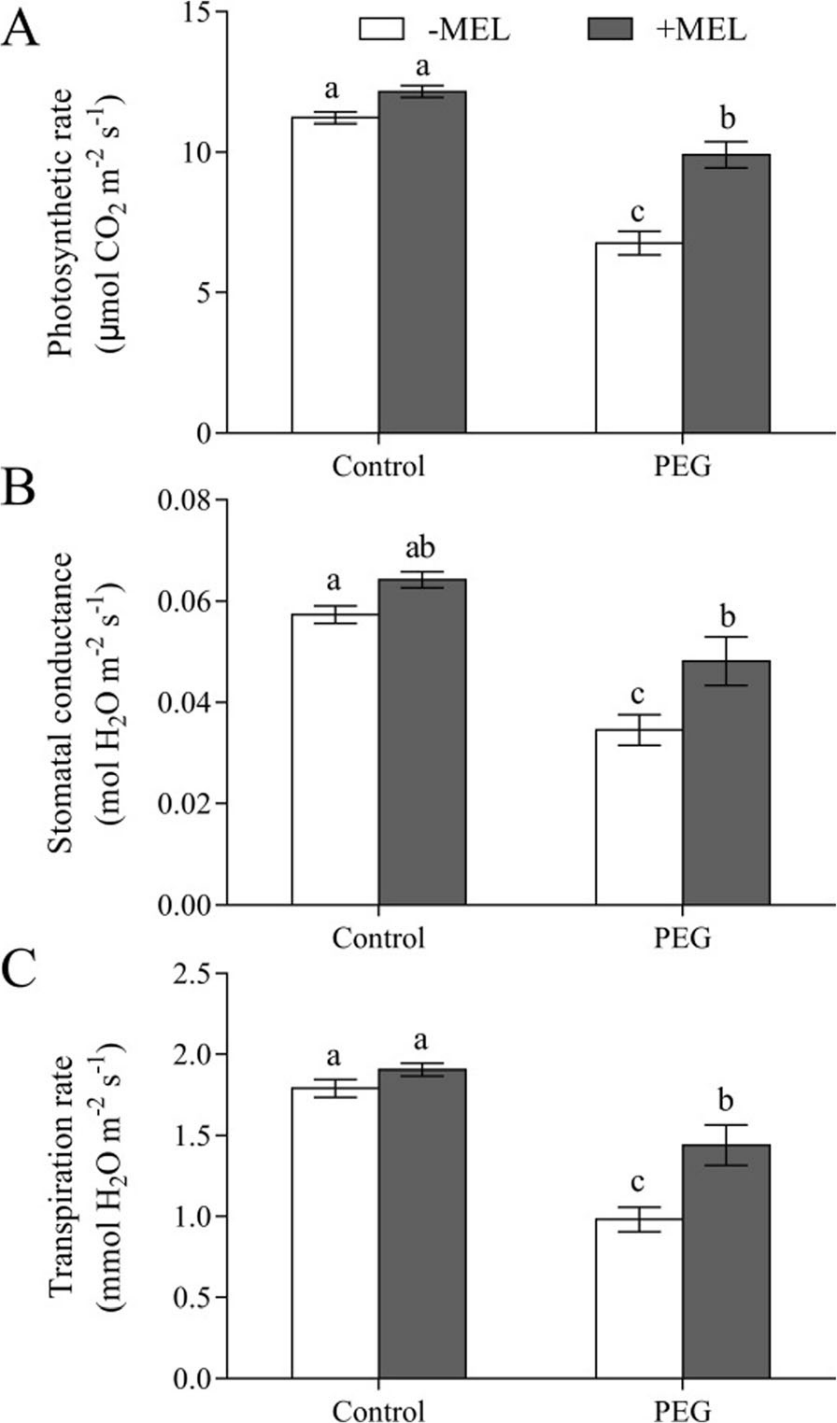
Effects of melatonin (MEL, 1 μM) application and water deficiency stress (PEG) on photosynthetic rate (**a**), stomatal conductance (**b**), and transpiration rate (**c**) of maize seedlings after 3 h of 10% PEG-6000 treatment. Values are presented as the means ± SE (*n* = 6). Different letters indicate statistically significant differences at *P* < 0.05

Under control condition, MEL application did not affect photosynthetic parameters in maize seedlings significantly. However, after exposed to water deficiency stress, all the photosynthetic parameters, including photosynthetic rate, stomatal conductance and transpiration rate, were reduced rapidly, but they were significant higher in MEL-treated seedlings than that without MEL-treated. Under water deficiency condition, the photosynthetic rate, stomatal conductance and transpiration rate were 46.6, 39.5 and 46.8% higher in MEL-treated seedlings than those without MEL treatment, respectively (Fig. 1).

### Application of MEL contribute to maintaining water status under water deficiency stress

**Fig. 2:**
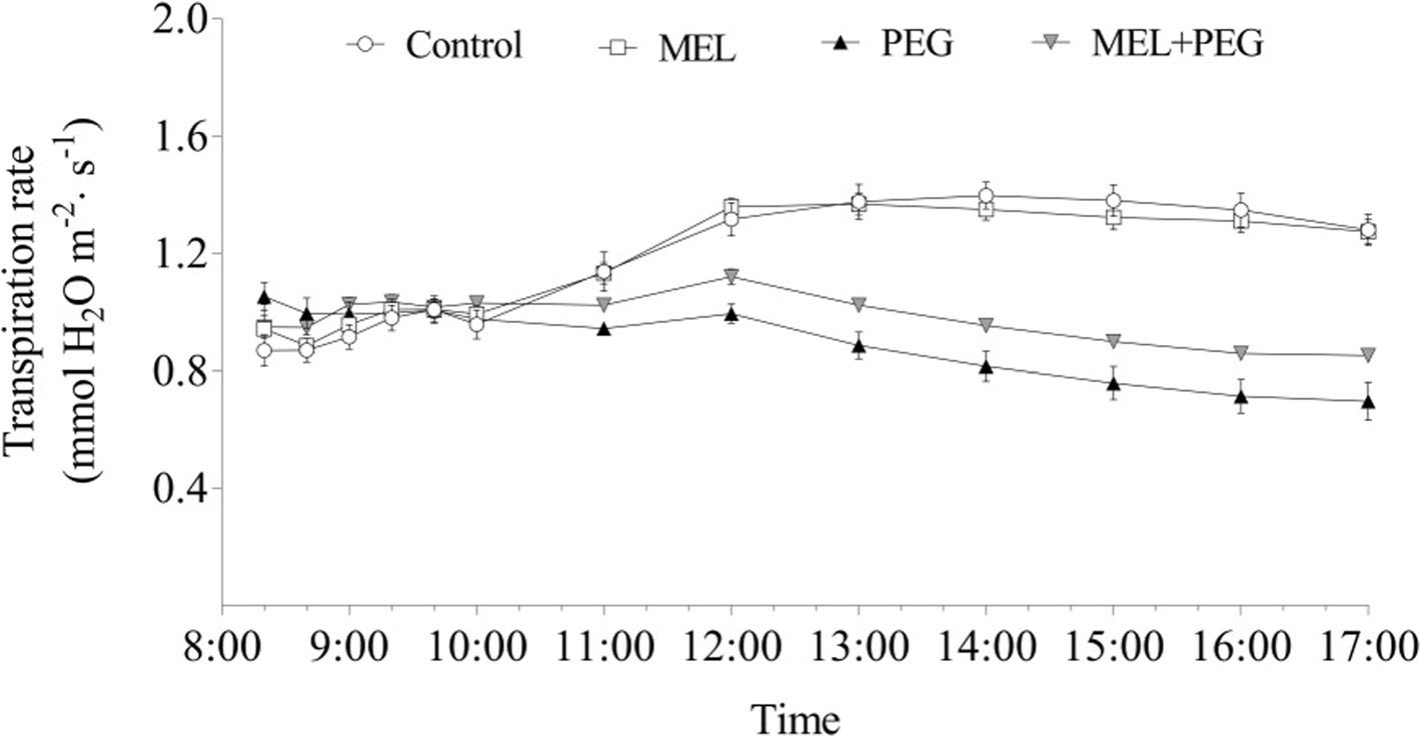
Effects of melatonin (MEL, 1 μM) application and water deficiency stress (PEG) on transpiration rate of maize seedlings in hydroponic culture. The transpiration rate was determined gravimetrically from the initial of PEG treatment at 08:00 a.m. (turn on the light). Values are presented as the means ± SE (*n* = 8). Different letters indicate statistically significant differences at *P* < 0.05

The whole plant transpiration rate was observed from the onset of PEG treatment to investigate the effect of exogenous MEL on the alteration of whole-plant water status under water deficiency condition. The transpiration rate fluctuated slightly at the beginning of the treatment and the significant difference began to occur after three hours of PEG treatment (11:00 a.m.), at which time point the whole-plant transpiration rate was higher in MEL-treated seedlings than that untreated, and this tendency was continued during the subsequent observation period (Fig. 2).

**Fig. 3:**
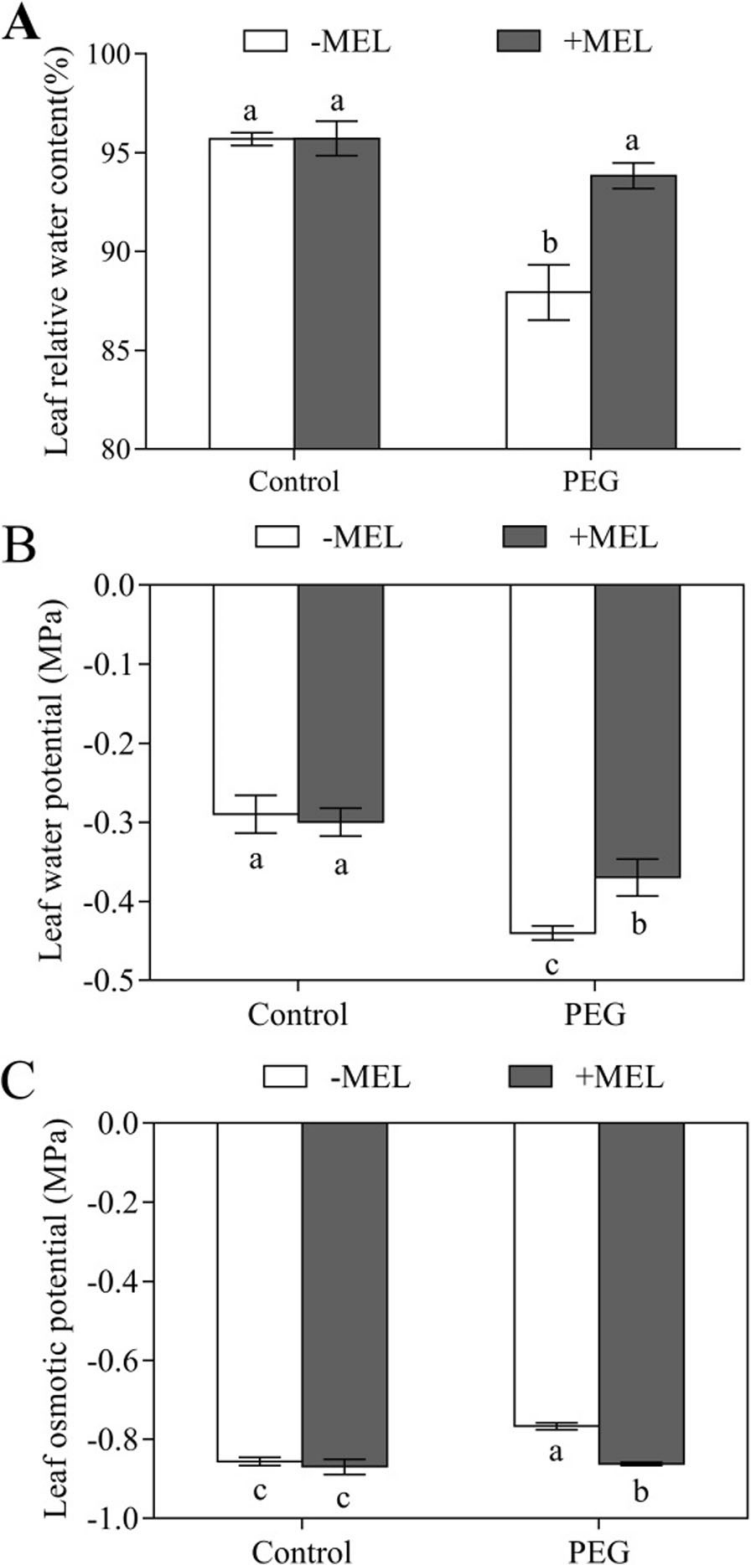
Effects of melatonin (MEL, 1 μM) application and water deficiency stress (PEG) on leaf relative water content (LRWC) (**a**), leaf water potential (**b**) and leaf osmotic potential (**c**) of maize seedlings in hydroponic culture. All parameters were determined after 3 h of PEG-6000 treatment. Values are presented as the means ± SE (*n* = 6). Different letters indicate statistically significant differences at *P* < 0.05

In the present study, the leaf relative water content (LRWC) did not change under control condition neither with nor without MEL application. However, it was decreased after three hours of water deficiency stress, while the MEL application significantly alleviated the stress-induced decrease in LRWC (Fig. 3a). After exposed to water deficiency stress for three hours, the leaf water potential in MEL-treated plants (− 0.37 MPa) also showed significantly higher than those of untreated (− 0.44 M Pa) (Fig. 3b). Furthermore, water deficiency stress significantly enhanced the leaf osmotic potential, but exogenous MEL reduced the water deficiency-induced increase in leaf osmotic potential, which showed 12.5% higher in MEL-treated seedlings than that without MEL treatment (Fig. 3c).

### Exogenous MEL improved the water status by maintaining a high K_plant_ and Lp_r_

**Fig. 4:**
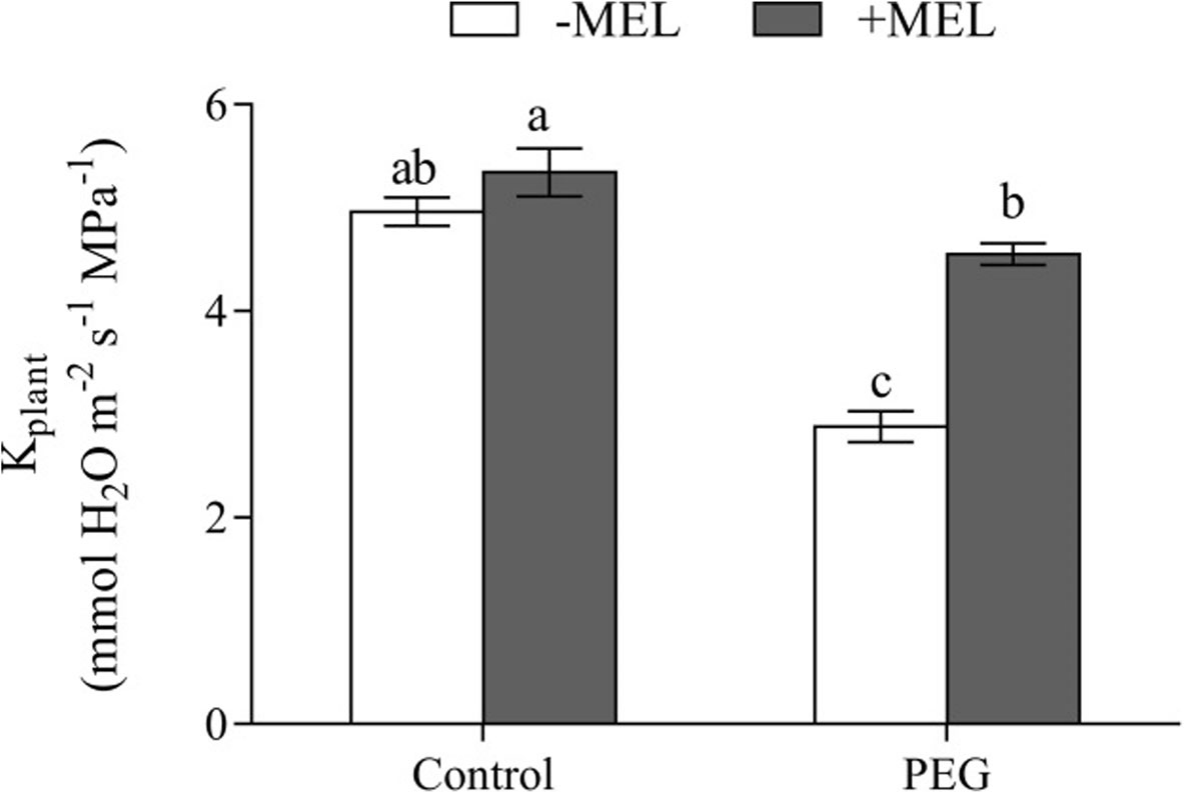
Effects of melatonin (MEL, 1 μM) application and water deficiency stress (PEG) on the whole-plant hydraulic conductance (K_plant_) of maize seedlings in hydroponic culture. The K_plant_ was calculated by the whole-plant transpiration rate divided by the difference between soil and leaf water potential. Values are presented as the means ± SE (*n* = 8). Different letters indicate statistically significant differences at *P* < 0.05

**Fig. 5:**
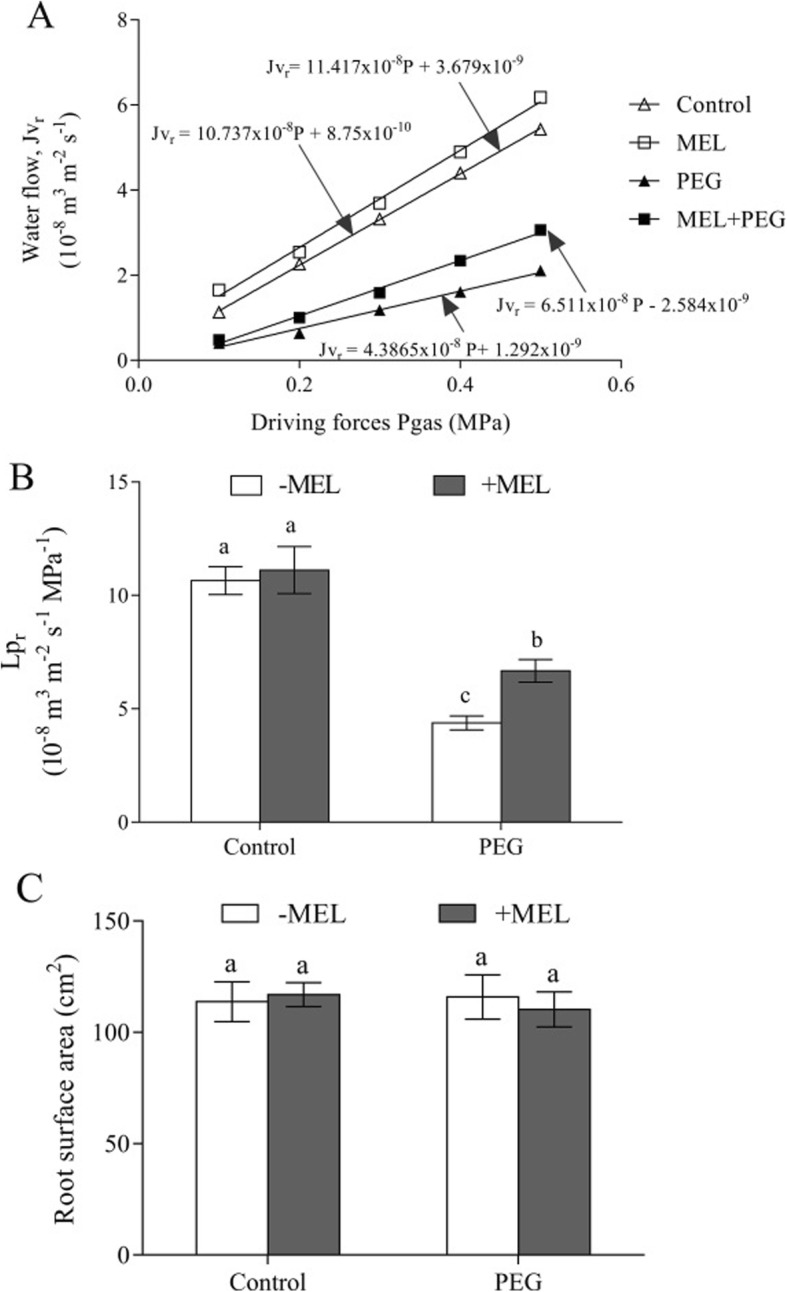
Effects of melatonin (MEL, 1 μM) application and water deficiency stress (PEG) on the water flow (Jv_r_) (**a**), root hydraulic conductance (Lp_r_) (**b**), and root surface area (**c**) of maize seedlings in hydroponic culture. Values are presented as the means ± SE (*n* = 6). Different letters indicate statistically significant differences at *P* < 0.05

Exogenous MEL had no obviously effect on the K_plant_ under control condition (Fig. 4). After exposure to PEG treatment, the K_plant_ was significantly decreased, but it was still higher in MEL-treated plants than that in MEL-untreated plants. The K_plant_ was 4.55 mmol H_2_O m^− 2^ s^− 1^ MPa^− 1^ in MEL-treated plants, which was 58% higher than that of untreated plants (2.88 mmol H_2_O m^− 2^ s^− 1^ MPa^− 1^). Similarly, the Lp_r_ also showed no significant difference regardless of MEL treatment under control condition (Fig. 5a, b). However, after exposed to PEG stress, the Lp_r_ of MEL-treated plants was 6.67 × 10^− 8^ m^3^m^− 2^s^− 1^MPa^− 1^, whereas it was only 4.37 × 10^− 8^ m^3^ m^− 2^ s^− 1^ MPa^− 1^ in MEL-untreated plants, which was 34.5% lower than that in MEL-treated plants. Furthermore, the short-term MEL treatment did not affect root surface area of maize seedlings, indicating that the enhanced root water uptake in MEL-treated plants could not be ascribed to the promoting of root growth (Fig. 5c).

### MEL application improved the whole-plant transpiration by increasing aquaporin activity

**Fig. 6:**
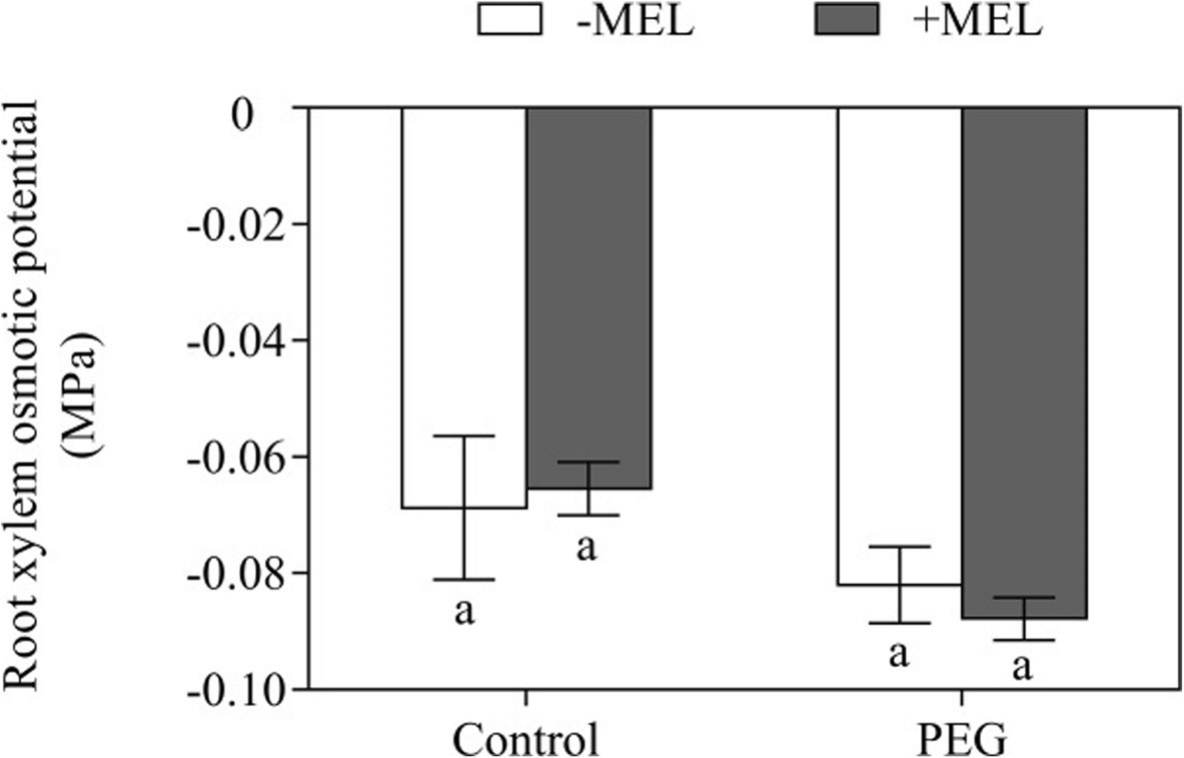
Effects of melatonin (MEL, 1 μM) application and water deficiency stress (PEG) on root xylem osmotic potential of maize seedlings in hydroponic culture. All parameters were determined after 3 h of PEG-6000 treatment. Values are presented as the means ± SE (*n* = 6). Different letters indicate statistically significant differences at *P* < 0.05

**Fig. 7:**
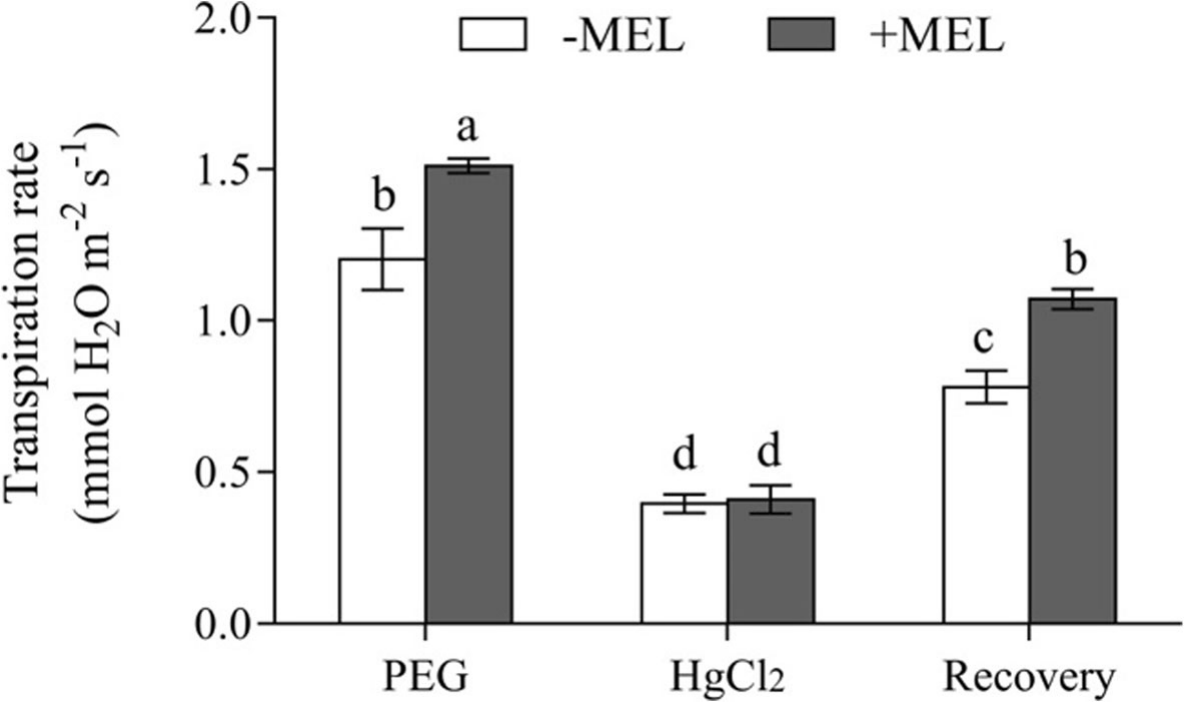
Effects of HgCl_2_ (aquaporin inhibitor) and DTT (dithiothreitol, anti-inhibitor) on the transpiration rate of MEL treated and untreated maize seedlings under water deficiency stress (PEG). After 3 h of PEG treatment, the seedlings were divided as follows: one group determined the transpiration rate directly, another group was treated with 50 μM HgCl_2_ for 5 min and then rinsed with distilled water before determining the transpiration rate, and the other was exposed to 50 μM HgCl_2_ (5 min) and 5 mM DTT (15 min) before measuring the transpiration rate. Values are presented as the means ± SE (*n* = 8). Different letters indicate statistically significant differences at *P* < 0.05

The osmotic potential of root xylem sap in the present study was not affect by MEL application under both control and PEG-stressed condition (Fig. 6). In the presence of HgCl_2_, the transpiration rate in MEL-treated and untreated plants decreased to the same level. After a recovery induced by DTT, the transpiration rate in MEL-treated plants were 37% higher than that of untreated (Fig. 7).

**Fig. 8:**
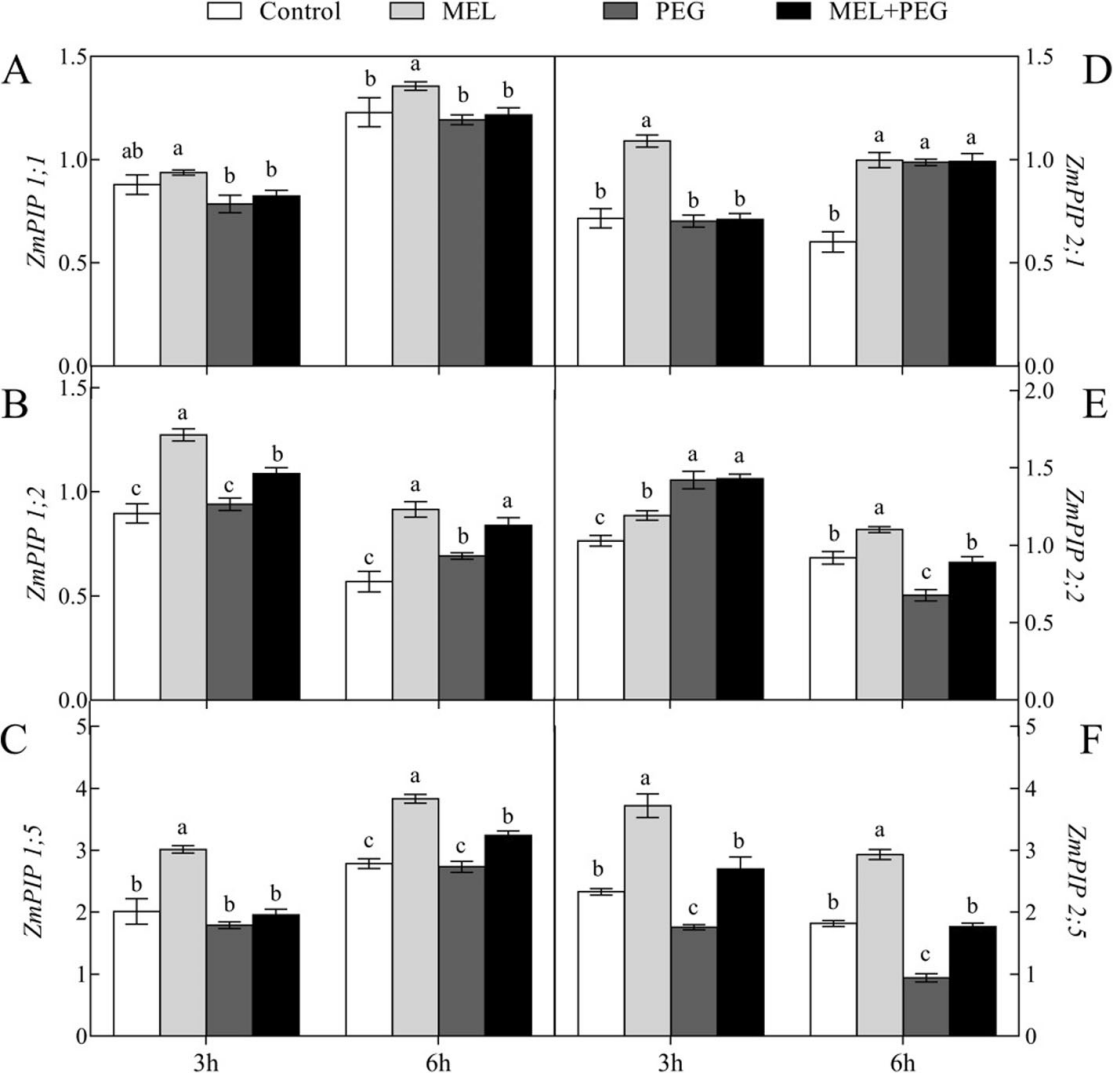
Effects of melatonin (MEL, 1 μM) application and water deficiency stress (PEG) on the root aquaporin gene expression level of maize seedlings in hydroponic culture. Root was sampled after 3 h and 6 h of PEG-6000 treatment. Values are presented as the means ± SE (*n* = 4). Different letters indicate statistically significant differences at *P* < 0.05

In addition, the expression levels of plasma membrane intrinsic aquaporins (PIPs) were up-regulated by MEL application under control condition. After 3 h of PEG treatment, the expressions of *ZmPIP1;2* and *ZmPIP2;5* were up-regulated by exogenous MEL. While after 6 h of PEG treatment, the expressions of *ZmPIP1;2*, *ZmPIP1;5*, *ZmPIP2;2* and *ZmPIP2;5* were all up-regulated by exogenous MEL (Fig. 8).

### Effects of MEL application on H_2_O_2_ content under water deficiency stress

**Fig. 9:**
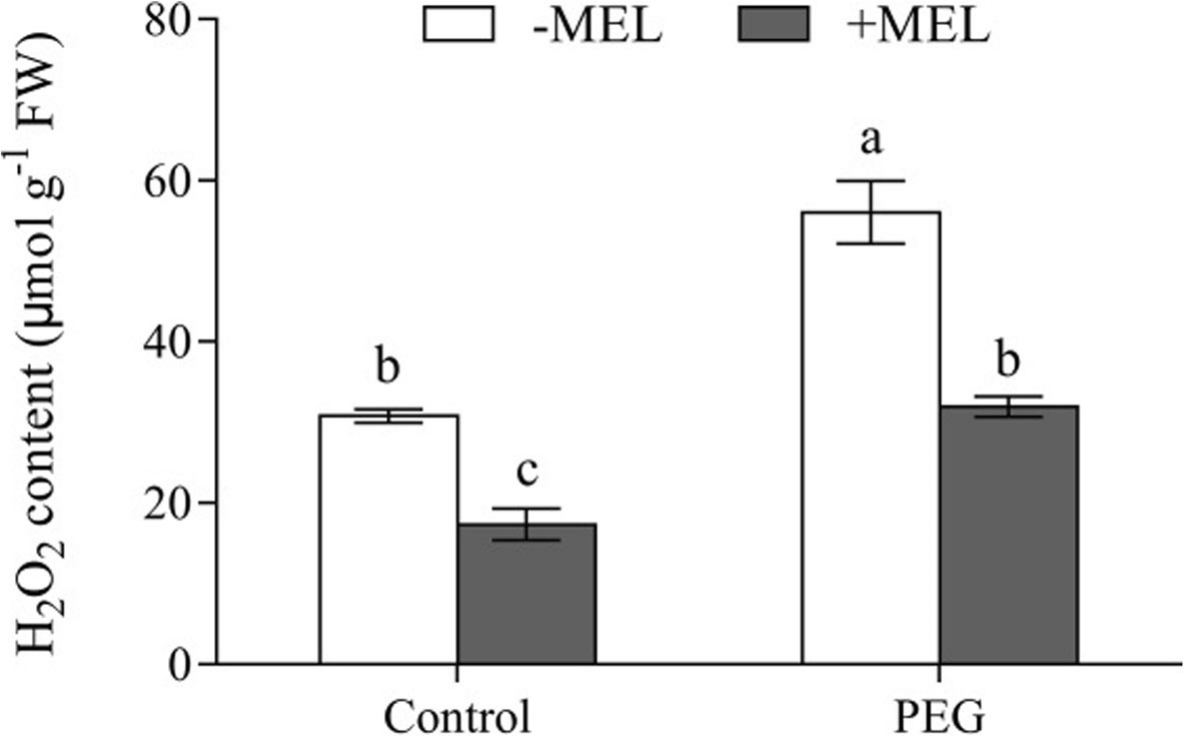
Effects of melatonin (MEL, 1 μM) application and water deficiency stress (PEG) on root H_2_O_2_ content of maize seedlings in hydroponic culture. H_2_O_2_ content was measured after 3 h of 10% PEG-6000 treatment. Values are presented as the means ± SE (*n* = 6). Different letters indicate statistically significant differences at *P* < 0.05

The H_2_O_2_ contents were lower in MEL-treated seedlings than that without MEL treatment under both control and water deficiency conditions. Moreover, the root H_2_O_2_ content increased sharply when exposed to PEG treatment, but it was kept at the same level as that in control plants (without MEL application) by MEL application (Fig. 9).

## Discussion

Water deficiency caused stomatal closure or destruction in photosynthetic reaction centers easily in plants, which can lead to the seriously decline of photosynthetic rate and ultimately affect the accumulation of dry matter [26, 27]. In the current study, our results showed that the water deficiency-induced reduction of photosynthetic rate was moderated in MEL-treated seedlings (Fig. 1), indicating that MEL application could alleviate plant water deficiency stress in maize seedling. Due to the short duration of MEL and PEG treatment, there was no visible differences in the dry weight between with and without MEL treatment (Fig. S1). In short term water deficiency stress, the main reason for the decrease of photosynthesis is caused by stomatal closure because of insufficient water supplied [28, 29]. In this study, compared with MEL-untreated plants, MEL-treated plants maintained high stomatal conductance, LRWC and transpiration rate under PEG treatment, suggesting that MEL could contribute to maintaining the water status of maize seedlings under water deficiency condition, which in turn favored in maintaining high transpiration and photosynthesis, and lead to improved plant water stress tolerance (Figs. 1, 2, 3). In addition, the influence of MEL on transpiration showed dose effect (Fig. S2).

The maintenance of plant water status depends on the coordinated balance among water absorption, transport and transpiration [30]. Ding et al. [31] have reported that exogenous application of MEL improved tomato tolerance to water deficiency by promoting cuticle formation to restrict water loss. In this study, due to the short duration of MEL and PEG treatment (only 24 h and 6 h, respectively), it is impossible for plant to reduce the loss of water by adjusting cuticle formation. Meanwhile, in this study, melatonin-treated plants maintained a relatively large stomatal conductance (Fig. 1b) and a higher whole plant transpiration rate (Fig. 2) under water deficiency stress. Such a large loss of water dispersion could not be ascribed to the water maintenance of the leaves under water deficiency stress, suggesting that MEL may alleviate the short-term water deficiency by increasing water absorption and transportation.

The K_plant_ represents the soil-to-leaf water transport capacity [21]. In this study, the K_plant_ was 58% higher in MEL-treated seedlings than that of untreated under water deficiency condition (Fig. 4), indicating that MEL could contribute to high water uptake capability under this condition. The K_plant_ consists of leaf (K_leaf_), stem (K_stem_) and root hydraulic conductance (Lp_r_) [21]. In previous studies, it has been clearly proved that the seedlings used in this experiment could not form stem due to the young age and short-term treatment [32], therefore, the K_plant_ can only be affected by K_leaf_ and Lp_r_. In this study, K_leaf_ was not measured because of the technical limitations. Brodribb et al. [33] showed that K_leaf_ is very similar to the leaf water potential, so we used leaf water potential to characterize the effect of MEL treatment on K_leaf_. Under water deficiency condition, MEL treatment significantly improved the water potential by 15.9% (Fig. 3). The Lp_r_ represents the root water uptake capacity, and its regulation plays an important role in maintaining the water status of the entire plant [34]. Under water deficiency condition, the Lp_r_ extremely limited the water uptake and transport [22]. In this study, after exposed to water deficiency stress, the Lp_r_ in MEL-treated plants was 52.5% higher than that of MEL-untreated plants. Meanwhile, under water deficiency condition, the increase of K_plant_ and Lp_r_ in MEL-treated plants exhibited the similar extent, which were 58 and 52.5% higher respectively (Figs. 4 and 5), suggesting that MEL could alleviate the water stress by regulating water uptake through improving the Lp_r_. The changing extent of K_plant_ is slightly higher than Lp_r_, suggesting that the high K_leaf_ in MEL-treated plants also contribute to the improvement of K_plant_ and the influence of the MEL on the K_leaf_ should also be investigated in the further study.

In root, radial water transport across cell membranes includes three pathways: apoplastic, symplastic and transcellular, and the latter two pathways together were called the “cell-to-cell” pathway [35]. Under normal growth condition (no water stress), plants absorb and transport water mainly through the apoplastic pathway, which is not regulated by aquaporins. In this study, under control condition, the MEL-treatment-induced up-regulation of aquaporin genes’ expression did not cause changes in root water absorption (Fig. 5). In addition, under normal condition, MEL treatment did not significantly affect the transpiration rate of maize seedlings (Fig. 2). Therefore, MEL treatment did not affect the water balance under normal water condition. Under abiotic stress, “cell-to-cell” pathway is the main water transport way, which is largely regulated by aquaporin [34, 36, 37]. Except for aquaporin, driving force of water transport, the root structure and its area could also influence the Lp_r_ [32, 38]. In our study, due to the short duration of MEL and PEG treatments, no visible change in root area and structure could be observed. Meanwhile, MEL treatment did not cause the change in xylem osmotic potential (Fig. 6). Taken together, aquaporin could be the main contributor for maintaining high Lpr in MEL-treated plant under water deficiency condition.

In the present study, aquaporin inhibitor and anti-inhibitor were used to investigate whether aquaporins are involved in regulation of plant hydraulic conductance has been widely reported previously [32, 39]. Here, HgCl_2_, a specific aquaporin activity inhibitor was used to eliminate the transpiration difference between MEL-treated and MEL-untreated plants. Under PEG treatment, the transpiration rates of seedlings were significantly higher in MEL treated plants than without MEL-treated plants. However, the transpiration rates in both MEL-treated and untreated plants were decreased to the same level after 5 min HgCl_2_ treatment. After recovery induced by DTT, the transpiration rates in MEL-treated seedlings were 37% higher than that measured in seedlings without MEL (Fig. 7). According to the homology and structural characteristics of amino acid sequences, plant aquaporins can be divided into Plasma membrane intrinsic aquaporins (PIPs), tonoplast intrinsic aquaporins (TIPs), nodulin 26-like aquaporins (NIPs), small basic intrinsic aquaporins (SIPs) [40]. TIPs are located on the vacuole membrane and mainly regulate the water transport between cytoplasm and vacuole. NIPs are located on the symbiotic membrane of rhizobia and legumes. SIPs are located on the endoplasmic reticulum membrane. Among these four types of aquaporins, PIP have shown to play the important role on the regulation of plant water homeostasis under water deficiency condition [21, 41]. Numerous studies have shown that the trafficking dynamics of PIPs is essential to improve root water absorption and leaf physiological conditions under drought stress [42, 43]. In this study, the transcript levels of aquaporin genes, including *ZmPIP1;2*, *ZmPIP1;5*, *ZmPIP2;2* and *ZmPIP2;5*, were significantly higher in MEL-treated plants than in untreated ones under both control and PEG treatment (Fig. 8). These results indicate that aquaporin activity was enhanced by MEL application which could contribute to the alleviation of water deficiency-induced decrease in Lp_r_. In this study, only the PIP transcript levels in the root were investigated, the whole aquaporins genes should be investigated both in root and leaf in the further study if the complex function of the aquaporins on the whole plant water balance is considered.

In this study, we found that MEL application enhanced the Lp_r_ by regulating the aquaporin activity, but the further molecular mechanism of this performance is not clear. Aquaporin activity is also affected by a number of plant hormones and regulators, such as salicylic acid, ethylene, Ca^2+^, and reactive oxygen species [44–46]. Although the molecular mechanisms of the MEL on the plant development and action were poorly understood, there are at least three approaches could be concluded based on the previous and current studies in animal and plants. Previous studies have largely shown that the accumulation of hydrogen peroxide can lead to the decrease of aquaporin activity [44, 47–50]. In the present study, the H_2_O_2_ levels in MEL-treated plants were significantly lower than that of untreated ones (Fig. 9), suggesting that MEL may be beneficial in maintaining aquaporin activity through reducing the accumulation of H_2_O_2_. On the other hand, the role of MEL in improving the antioxidant activity has also been considered as the main function for MEL to improve plant stress tolerance [1, 10, 18]. Therefore, MEL may regulate aquaporin activity through scavenging of hydrogen peroxide. Studies in animals have shown that exposure to MEL can induce an increase in intracellular cyclic adenosine monophosphate levels [51]. Upon cyclic adenosine monophosphate elevation, it will cause a decrease of phosphorylation and poly-ubiquitination and hence facilitates the trafficking aquaporin bearing vesicles to the plasma membrane [52]. In addition, more evidence showed that there is significant crosstalk between MEL and other plant hormones, including: cytokinin, salicylic acid, jasmonic acid, gibberellins, abscisic acid and ethylene [2, 50–55]. Although no direct evidence has been shown up to date that MEL could regulate aquaporin activity by affecting plant hormones or signaling molecules, considering the extensive interaction between MEL and plant hormones, the possibility that MEL regulates aquaporin activity through plant hormones or signaling molecules cannot be ignored.

## Conclusions

**Fig. 10:**
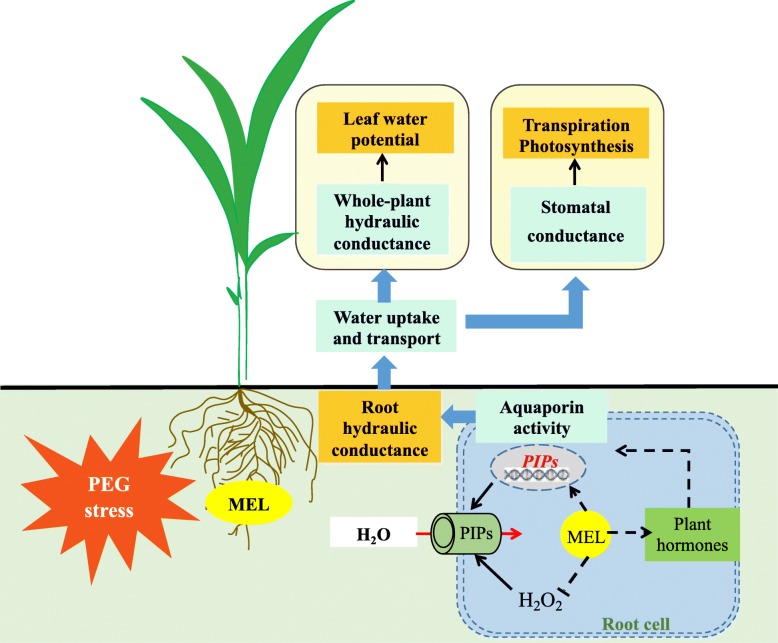
A schematic model for melatonin (MEL) alleviates PEG-induced short-term water deficiency in maize by improving the water uptake and transport. MEL may enhance the aquaporin activity by two ways: (1) up-regulating the expression of aquaporin genes and alleviating the decrease of aquaporin activity caused by H_2_O_2_; (2) potential interacting with plant hormones. The enhanced aquaporin activity resulted in increased root hydraulic conductance, which contributed to an increase in water uptake and transport, thereby maintaining the high leaf water potential, stomatal conductance, and photosynthetic rate, and enhancing the tolerance to water deficiency

Numerous studies have shown that MEL plays an important role in plants to improve various environmental stress tolerance. This study showed that exogenous MEL improves plant water deficiency tolerance by regulating water absorption. Based on previous and our current study, the potential mechanism diagram of MEL on alleviating short-term water deficiency stress was proposed. (1) Under PEG treatment, MEL application enhanced aquaporin activity with decreased H_2_O_2_ accumulation and upregulated the transcription of *ZmPIP* genes. (2) The performance of PEG-induced decrease in Lp_r_ and K_plant_ were mitigated by the high aquaporin activity. (3) The high K_plant_ was beneficial to maintaining the high leaf water content, stomatal conductance, and photosynthetic rate, thereby, enhancing the tolerance to water deficiency stress (Fig. 10). In addition, the underlying mechanisms by which MEL promotes the up-regulation of aquaporin gene expression remain unclear, and future work should be carried out from the aspects of intrinsic molecular mechanisms or signaling pathways of MEL on regulation of plant hydraulic conductance. Moreover, under water-deficient condition, plants could also maintain water balance by increasing root water uptake and/or reducing leaf water loss. Since our current study is a short-term water stress, how MEL regulates plant water balance from the aspects of water uptake and loss under long-term water stress condition still needs more research.

## Methods

### Plant materials, growth condition and MEL treatment

The commercial hybrid maize seeds [*Zea mays* (L.), cv. Zhengdan958] were bought from Shandong Ruiyou Agriculture and Technology Development Company Limited (Jinan City, Shandong Province, China), and this cultivar is a widely grown cultivar in China. The experiment was carried out in a growth chamber which was set to a 14/10 h day/night cycle at the temperature of 28/25 °C with 40 to 50% relative humidity. The amount of photosynthetically active radiation at the upper plant was 500 μmol m^− 2^ s^− 1^. Seeds of maize were germinated on two layers of moist filter paper for 3 days in an incubator under dark conditions at 28 °C. After germination, uniform seedlings (6 ± 0.5 cm high) were selected and transplanted into a plastic container (40× 28× 14 cm) with 5 l of half strength Hoagland culture solution for growing. After six days of transplanting, the plants were divided into two parts for 0 μM or 1 μM MEL treatment, respectively. The culture solution was continuously aerated, and the pH was adjusted to 6.0 with 0.1 M HCl or 1 M KOH every day. Twenty-four hours after MEL treatment, 10% PEG-6000 was added at 8:00 a.m. for simulating water deficiency stress. The PEG treatment was added and lasted for 10 h. Thus, four treatments: Control, MEL, PEG and MEL+PEG were included in this experiment.

### Photosynthetic rate, stomatal conductance, and transpiration rate

Three hours after PEG treatment, the photosynthetic rate, stomatal conductance, and transpiration rate were measured with a portable photosynthesis system (Li-6400; LI-COR Inc., Lincoln, NE, USA). The top fully expanded leaf was placed in a 6 cm^2^ chamber at a photo flux density of 500 μmol m^− 2^ s^− 1^ with leaf temperature was 28 °C.

### Whole-plant transpiration rate

The transpiration rate was measured by gravimetric method according to Liu et al. [32] from the onset of PEG treatment. The seedlings were placed in a foil-wrapped plastic bottle containing the original culture solution, and sealed with a sealing film to prevent water dispersion. The rate of water dispersion per unit time was determined using a balance. It was measured every 20 min during the first two hours and then measured every one hour until the end of PEG treatment. The leaves were scanned with a scanner and the leaf area of each plant was calculated using image analysis software (WinRHIZO PRO 2009, Canada). The whole plant transpiration rate was calculated by dividing the rate of water dispersion per unit time by the corresponding leaf area. Six replicates were included for each treatment.

### Leaf relative water content, leaf water potential and osmotic potential

The upper fully expanded leaves were used for leaf relative water content (LRWC) measurement after three hours of PEG treatment according to Liu et al. [32]. The leaf was cut into 2 cm segments with scissors, and the fresh weight was quickly weighed with the balance. The leaf segments were placed in a 50 mL centrifuge tube and filled with distilled water for 6 h, and then the total weight was measured after drying the water. Then, after drying at 70 °C for more than 48 h, the dry weight was weighed. The LRWC was calculated as: LRWC = [(Fresh weight -Dry wight)/ (Total weight -Dry weight)] × 100%.

The leaf water potential was measured according to Chen et al. [39] by a pressure chamber (Model 3500, Soil Moisture Corp., Santa Barbara, CA, USA). Leaf was cut off in the middle and placed into the steel drum with the slit exposed about 1 cm. After tightening the spiral collar, opened the main control valve and slowly pressurized at a rate of 0.05 MPa per second. When the water film appeared in the slit, the pressure value was the water potential value of the leaf.

The leaf osmotic potential was measured as follows: The latest fully expanded leaves were shredded, mixed and inserted into a centrifuge tube which was previously punched at the bottom. After freezing in the liquid nitrogen, store at − 20 °C. Frozen leaf samples were thawed at room temperature, then centrifuged at 4000 rpm for 5 min to gather the cell sap and the cell sap was measured using a dew point microvolt meter (Model 5600, Wescor, Logan, UT, USA). Each treatment included six replicates.

### Osmotic potential of root xylem

The osmotic potential of the root xylem sap was measured after three hours of PEG treatment according to the method of Liu et al. [32]. The shoot was cut off at the base of the root system and the xylem sap was collected using a pressure chamber (Model 3500, Soil Moisture Corp., Santa Barbara, CA, USA). The entire root system was sealed in the cylinder leaving 1 cm of mesocotyl exposed outside the rubber stopper. After tightening the spiral collar, opened the main control valve and applied a pressure less than the leaf water potential to cause the xylem sap to flow out of the incision. The xylem sap was collected in a 0.5 ml centrifuge tube and its osmotic potential was measured using a dew point microvolt meter as mentioned above. Each treatment included six replicates.

### Whole-plant hydraulic conductance (K_plant_)

The K_plant_ was calculated according to the following equation [21]: K_plant_ = Transpiration rate / (Soil water potential -Leaf water potential). The transpiration rate refers to the whole plant level determined gravimetrically. In hydroponic culture, the soil water potential (i.e. culture potential) was measured using a dew point microvolt meter as used above. In detail, the culture potential was − 0.03 MPa under control condition or with MEL application. Under PEG treatment, the soil water potential (i.e. culture potential) was − 0.19 MPa. The leaf water potential was measured by a pressure chamber as introduced before. Each treatment included eight replicates.

### Root hydraulic conductance and root surface area

The root hydraulic conductance (Lp_r_) was measured after three hours of PEG treatment with a pressure chamber (as mentioned above) according to the method of Miyamoto et al. [55]. Each shoot was cut off at the base of the root system leaving 1 cm of mesocotyl and the roots were enclosed in a steel chamber. The gas pressure (P_ages_) in the chamber was raised in steps of 0.1 MPa up to 0.5 MPa and under each given pressure, the exuded sap was collected with absorbent cotton for 60s and weighed. After that, the root surface area was determined using a scanner and analyzed by root image analysis software (WinRHIZO PRO 2009, Regent Inc., Canada). The water flow (Jv_r_) in m^3^ m^− 2^ s^− 1^, is the slopes of exuded sap weight and time referred to the unit root surface area. Lp_r_ is calculated from the slopes of Jv_r_ against driving force. Lp_r_ was determined according to the following equation: Jv_r_ = Lp_r_ × P_gas_. Each treatment included six replicates.

### Transpiration rate in response to aquaporin inhibitor (HgCl_2_) and anti-inhibitor (DTT)

The changes in the transpiration rate in response to aquaporin inhibitor (HgCl_2_) and anti-inhibitor (DTT) were used to investigating the aquaporin-mediated water transport according to the method of Liu et al. [32]. The seedlings were divided as follows: one group was used for determining the transpiration rate directly, another group was treated with 50 μM HgCl_2_ for 5 min and then rinsed with distilled water before determining the transpiration rate, and the third group was exposed to 50 μM HgCl_2_ for 5 min and then treated with 5 mM DTT for 15 min. After that, roots were washed with distilled water, then the whole-plant transpiration rate was determined gravimetrically. Each treatment included eight replicates.

### Expression levels of maize aquaporin genes

Roots tips (5 cm) were sampled after three and six hours of PEG treatment, respectively. The expression levels of eight identified maize aquaporin genes were analyzed using quantitative real-time -PCR. Total RNA was extracted by a TakaRa MiniBEST Plant RNA Extraction Kit (TakaRa, Dalian, China) and cDNA synthesis was reverse transcribed by a PrimeScriptTM II 1st Strand cDNA Synthesis Kit (TakaRa, Dalian, China). Quantitative real-time PCR analysis was conducted by a LightCycler 480 II System (Roche, Basel, Switzerland) using a SYBR Premix Ex Taq™ kit (TakaRa, Dalian, China). The relative expression levels of *ZmPIPs* were assessed using glyceraldehyde phosphate dehydrogenase (*GAPDH*) as the internal standard. To confirm the specificity of primers to the target genes, normal PCR was run. Each treatment included three replicates and each replicate included three technical replicates. The genes and the sequences of their specific primers are presented in Table 1.

**Table 1:** Primers of aquaporin genes and reference gene used in real time PCR experiments

Gene	Primer
*ZmPIP1;1*	cccctactatgttacgtggagttc
	gcggcatattacacaattggta
*ZmPIP1;2*	ctcattttatgcgttgggatgt
	actgaaaccaagaaaaccctga
*ZmPIP1;5*	cacgtggtcatcatcaggg
	cgtatgctgcatggttgct
*ZmPIP2;1*	cgggtcgccttttttttg
	cccttgagagtcacgacatga
*ZmPIP2;2*	ggccttctaccaccagtacatc
	ggcctttctttagctctgctc
*ZmPIP2;5*	tgtcgtcgttggttgcct
	cacaacaatcacactagcttggaa
*GAPDH*	agcaggtcgagcatcttcg
	ctgtagccccactcgttgtc

### H_2_O_2_ content measurement

Three hours after PEG treatment, the roots of maize seedlings were sampled to measure the H_2_O_2_ content according to the method of Ryan et al. [56]. Root tissues (0.3 g) were ground into fine powder and homogenized with 2 mL cold 0.1% (w/v) trichloroacetic acid. After centrifuged at 12,000 g for 30 min at 4 °C, 0.4 mL of the supernatant was mixed with 0.4 mL 10 mM potassium phosphate buffer (pH 7.0) and 0.8 mL 1 M KI. The absorbance of the mixture was read at 390 nm, and the content of H_2_O_2_ in the sample was calculated against a calibration curve using H_2_O_2_ standards. Each treatment included four replicates from four different individual plants.

### Statistical analysis

All experiments were repeated at least twice. Data were statistically analyzed using Duncan test (*p* < 0.05) with SPSS 19.0 (IBM, USA). All plots were created using GraphPad Prism 7.00 and different letters indicated statistically significant differences at *p* < 0.05.

## Supplementary information

**Additional file 1: Figure S1.** Effects of melatonin (MEL) application and water deficiency stress (PEG) on the dry weight of maize seedlings in hydroponic culture. **Figure S2.** Effects of melatonin (MEL) application and drought stress on transpiration rate of maize seedlings in hydroponic culture.

## Data Availability

The data used in this study is available from the corresponding author on reasonable request.

## Abbreviations

PEG: Polyethylene glycol
MEL: Melatonin
K_plant_: Whole-plant hydraulic conductance
Lp_r_: Root hydraulic conductance
DTT: Dithiothreitol
LRWC: Leaf relative water content
K_leaf_: Leaf hydraulic conductance
K_stem_: Stem hydraulic conductance
Pages: Gas pressure
Jvr: the water flow
GAPDH: Glyceraldehyde phosphate dehydrogenase
PIPs: plasma membrane intrinsic aquaporins (PIPs)
TIPs: Tonoplast intrinsic aquaporins
NIPs: Nodulin 26-like aquaporins (NIPs)
SIPs: Small basic intrinsic aquaporins
