# Supplementary material for: Exogenous melatonin alleviates PEG-induced short-term water deficiency in maize by increasing hydraulic conductance
Source: BMC Plant Biol. 2020 May 14;20:218. doi: 10.1186/s12870-020-02432-1 (PMC7227339; doi:10.1186/s12870-020-02432-1)
Supplement: Supplementary file 1 — Additional file 1: Figure S1. Effects of melatonin (MEL) application and water deficiency stress (PEG) on the dry weight of maize seedlings in hydroponic culture. Figure S2. Effects of melatonin (MEL) application and drought stress on transpiration rate of maize seedlings in hydroponic culture. [file 12870_2020_2432_MOESM1_ESM.docx]

Additional Figure


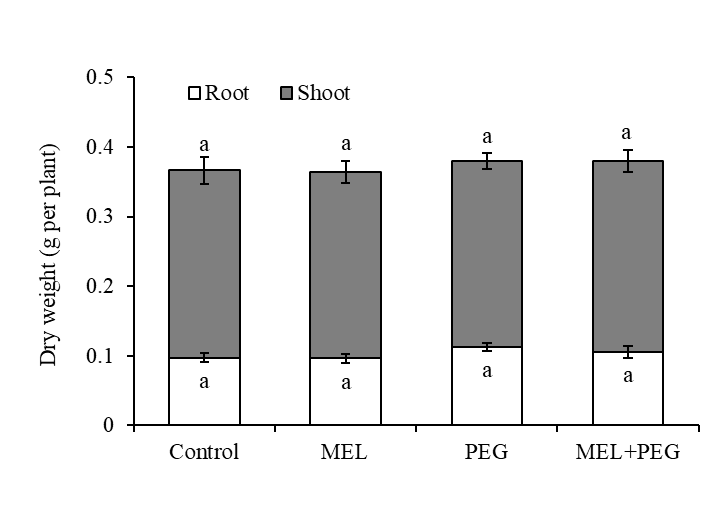


Additional Fig. S1 Effects of melatonin (MEL) application and water deficiency stress (PEG) on the dry weight of maize seedlings in hydroponic culture. All parameters were determined after 3h of PEG-6000 treatment. Values are presented as the means ± SE (n=8). Different letters indicate statistically significant differences at P< 0.05.


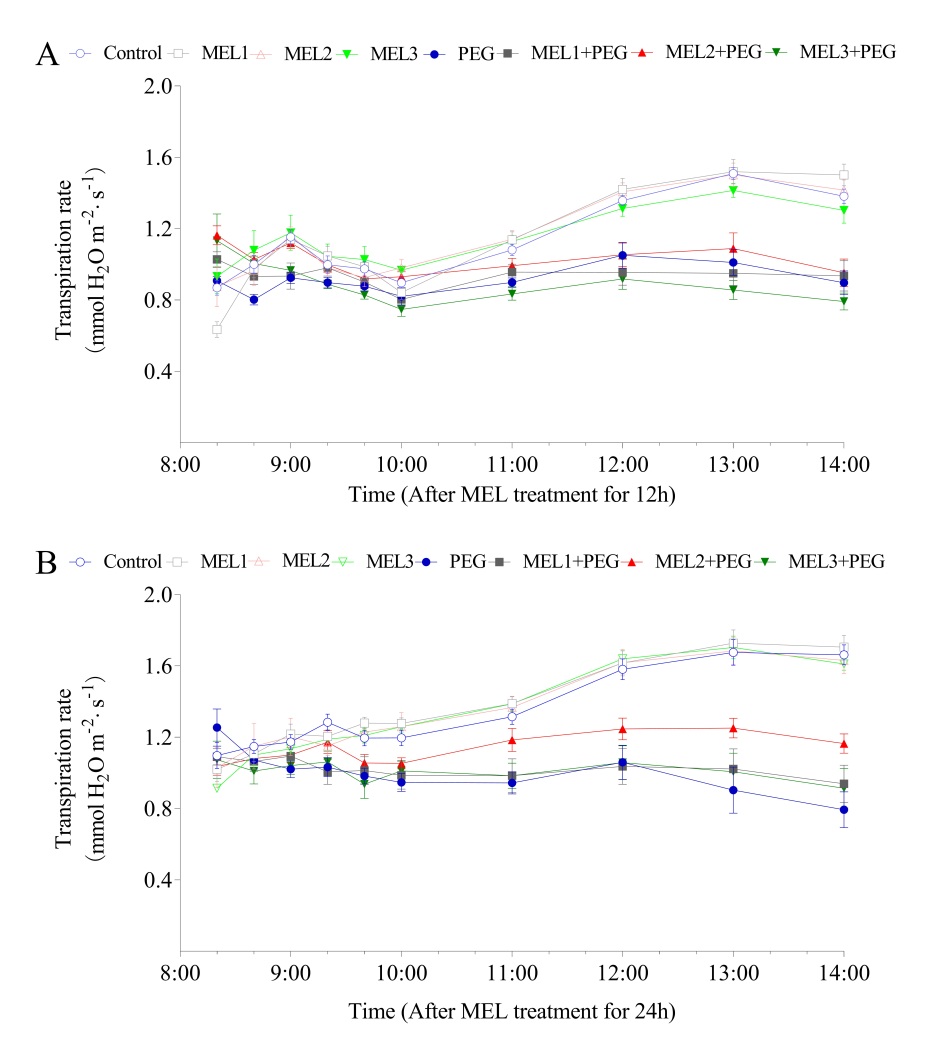


Additional Fig. S2 Effects of melatonin (MEL) application and water deficiency stress (PEG) on transpiration rate of maize seedlings in hydroponic culture. Melatonin treatment settings are as follows: 0 μM (control), 0.1 μM (MEL1), 1 μM (MEL2), 10 μM (MEL3). After 12 hours (A) and 24 hours (B) MEL treatment, 10% PEG-6000 (–0.19 MPa) was added at 08:00 a.m. to induce osmotic stress. The transpiration rate was determined gravimetrically from the initial of PEG treatment at 08:00 a.m. (turn on the light). Values are presented as the means ± SE (n=8). Different letters indicate statistically significant differences at P<0.05.
